# A Virulence Factor Encoded by a Polydnavirus Confers Tolerance to Transgenic Tobacco Plants against Lepidopteran Larvae, by Impairing Nutrient Absorption

**DOI:** 10.1371/journal.pone.0113988

**Published:** 2014-12-01

**Authors:** Ilaria Di Lelio, Silvia Caccia, Mariangela Coppola, Martina Buonanno, Gennaro Di Prisco, Paola Varricchio, Eleonora Franzetti, Giandomenico Corrado, Simona M. Monti, Rosa Rao, Morena Casartelli, Francesco Pennacchio

**Affiliations:** 1 Dipartimento di Agraria, Università di Napoli “Federico II”, Portici, Italy; 2 Istituto di Biostrutture e Bioimmagini (IBB), CNR, Napoli, Italy; 3 Dipartimento di Scienze e Tecnologie Ambientali, Biologiche e Farmaceutiche, Seconda Università di Napoli, Caserta, Italy; 4 Dipartimento di Bioscienze, Università degli Studi di Milano, Milano, Italy; Institute of Plant Physiology and Ecology, China

## Abstract

The biological control of insect pests is based on the use of natural enemies. However, the growing information on the molecular mechanisms underpinning the interactions between insects and their natural antagonists can be exploited to develop “bio-inspired” pest control strategies, mimicking suppression mechanisms shaped by long co-evolutionary processes. Here we focus on a virulence factor encoded by the polydnavirus associated with the braconid wasp *Toxoneuron nigriceps* (*Tn*BV), an endophagous parasitoid of noctuid moth larvae. This virulence factor (*Tn*BVANK1) is a member of the viral ankyrin (ANK) protein family, and appears to be involved both in immunosuppression and endocrine alterations of the host. Transgenic tobacco plants expressing *Tn*BVANK1 showed insecticide activity and caused developmental delay in *Spodoptera littoralis* larvae feeding on them. This effect was more evident in a transgenic line showing a higher number of transcripts of the viral gene. However, this effect was not associated with evidence of translocation into the haemocoel of the entire protein, where the receptors of *Tn*BVANK1 are putatively located. Indeed, immunolocalization experiments evidenced the accumulation of this viral protein in the midgut, where it formed a thick layer coating the brush border of epithelial cells. *In vitro* transport experiments demonstrated that the presence of recombinant *Tn*BVANK1 exerted a dose-dependent negative impact on amino acid transport. These results open new perspectives for insect control and stimulate additional research efforts to pursue the development of novel bioinsecticides, encoded by parasitoid-derived genes. However, future work will have to carefully evaluate any effect that these molecules may have on beneficial insects and on non-target organisms.

## Introduction

The protection of crop plants and of their products against insects has old roots, dating back to the origin of agriculture. Insect control has been for a long time handled with little use of chemicals, until when a growing number of synthetic molecules [1], often with neurotoxic properties, reached the market, providing the illusory perception that all pest management problems would have been solved just with the use of chemical pesticides. Indeed, the insecticides have been, and still are, an important tool in intensive agriculture [2], [3], but the unfortunate misuse of these substances has evidenced, over the years, the limits and the poor sustainability of this simplistic approach. This has promoted research efforts toward the development of more benign insecticide molecules of natural origin [4] and of integrated pest management (IPM) strategies, more sustainable both from an ecological/toxicological and economic point of view [5], [6], from field level to a broader spatial scale [7]. Moreover, this trend has also boosted the definition of control strategies based on the use of natural antagonists, which has favored the successful consolidation of classical biological control [8].

The continuous growth of basic studies on the antagonistic associations between insects and their natural enemies has generated background information on the underpinning molecular interactions, which offers the opportunity to develop “bio-inspired” pest control strategies, mimicking natural processes. There are already very good examples of new pest control tools generated by this nature-driven approach, which include, for example, the well consolidated use of the entomopathogen *Bacillus thuringiensis* and its derived toxins, both for direct application and for developing transgenic crops [9]. Similarly, molecules like spinosyns [10], [11] are obvious examples of natural insecticides of wide use in IPM, while a number of natural compounds, of plant origin [12] or produced by predatory arthropods [13], are increasingly used or look particularly promising for future developments.

There is no doubt that the impressive diversity of Hymenoptera, which represent the largest group of insect antagonists and include nearly 10–20% of all insects [14], [15], is the largest reservoir of molecular biodiversity, that can offer a wide selection of virulence factors, having potential insecticide activity against a number of insect species. The host-parasitoid associations in basal evolutionary lineages are characterized by the presence of venom blends which contain active components causing rapid and irreversible paralysis, used by idiobionts to block the development of their hosts, while more derived lineages, often showing endophagy, are koinobionts that regulate a number of physiological traits of their hosts, which continue to grow and are not paralyzed/suppressed [16]. Among these latter, there is a group of ichneumonoids, endophagous parasitoids of lepidopteran larvae, which harbor a symbiotic virus in the family Polydnaviridae [17]–[19]. Polydnaviruses (PDV) are among the most potent viral immunosuppressors existing in nature and encode host regulation factors which are able to modulate host physiology, by disrupting its vital functions, in order to create a suitable environment for the development of parasitoid's progeny [16]. Briefly, these viruses are integrated as proviruses in the wasp genome, which into the ovary generate free viral particles, injected in the host body along with the egg and venom. Then, the virions infect host tissues, where they express several virulence factors, without undergoing replication. The genome of PDV contains a selection of sequences encoding molecules able to disrupt the physiology, development and reproduction of insect hosts, and, therefore, represents a unique source of natural molecules with potential insecticide activity.

This natural reservoir of putative bioinsecticide molecules remains largely unexploited. Indeed, there are just a few examples in the literature of studies focusing on the use of parasitoid-derived molecules to develop new pest control strategies. Among these, we have the successful attempt of using the coding genes of Cys-motif proteins to generate transgenic plants, which showed a significant level of protection against lepidopteran larvae [20], [21]. These studies demonstrated that molecules derived from parasitic wasps and their associated viral symbionts are active when orally administered, despite of the fact that they are expected to exert their activity on receptors located in the haemocoel, often at intracellular level. Even though the absorption of large macromolecules by the gut epithelium of insect is possible, and can be enhanced by different molecular strategies [22], these pioneering studies did not provide any evidence on how and where (*i.e.* in the gut or behind the gut barrier) the virulence factors used were able to exert their activity.

We have contributed to the study of the functional and molecular interactions between the braconid wasp *Toxoneuron nigriceps* and the larvae of its natural host, *Heliothis virescens*, shedding light on the role played in the host regulation process by the associated bracovirus (*Tn*BV) (reviewed in [16]). Among the different virulence factors encoded by *Tn*BV, we have focused our attention, over the past number of years, on the functional analysis of a gene family encoding viral ankyrin proteins (ANK), which are largely shared by many PDV, both in the bracoviruses and ichnoviruses [19]. In particular, we have studied the immunosuppressive activity and the potential role as endocrine disrupter of *Tn*BVANK1 [23]–[25]. The wide distribution in different parasitoid lineages and the pleiotropic effects of these ANK proteins stimulated the idea of exploring their potential use as disrupters of lepidoteran larvae physiology, in order to identify potential candidate molecules to be further developed as novel bioinsecticides.

Here we provide evidence showing that *Tn*BVANK1, when expressed in transgenic tobacco plants, has significant insecticide activity. To understand how this insecticide activity is exerted, we investigated the fate of the ingested molecule and its possible mechanism of action.

## Material and Methods

### 2.1 Molecular cloning

MycKDEL sequence was produced by annealing specific oligonucleotides (5′-CTAGAATGGAGCAAAAGCTCATTTCTGAAGAGGACTTGAAAGATGAACTGTAAG-3′; 5′-GATCCTTACAGTTCATCTTTCAAGTCCTCTTCAGAAATGAGCTTTTGCTCCATT-3′). These oligonucleotides were mixed in equal concentrations, boiled for 5 min and then allowed to reach room temperature. The fragment obtained was inserted into pDE::SP vector, deriving from pAmy [26]. *Tn*BV*ank1* cDNA (accession number AJ583457) was amplified using specific primers: Forward: 5′-GCTGGTACCAATGGAAAACTCATTACTCATTG-3′; Reverse: 5′-CCTCGAGATATCATTATCATCACACTTAGCGC-3′ (the underlined sequences, containing KpnI and EcoRV restriction endonucleases targets respectively, were added for the subsequent cloning), then cloned into pCR1.2 plasmid (TA cloning, Invitrogen), and later transferred into pDE::SP vector, along with the MycKDEL sequence, in order to obtain the pDE::Sp-ank1-MycKDEL. *Escherichia coli* DH5α cells were transformed via thermal shock and checked by colony PCR. pDE::Sp-ank1-MycKDEL plasmid was digested, using *Eco*RI and *Hind*III enzymes, in order to extract the expression cassette that was ligated into PG0029 binary vector. *Agrobacterium tumefaciens* LBA4404 cells were transformed, using 1 µg of PG0029::Sp-ank1-MycKDEL and 1 µg of the pHelper plasmid pSOUP through thermal shock, and streaked on selective medium.

### 2.2 Plant transformation

For transformation experiments, *Nicotiana tabacum* ‘Samsun’ NN tobacco plants were grown under sterile conditions from seeds. *A. tumefaciens-*mediated leaf disk tobacco transformation and regeneration of antibiotic-resistant plants were performed according to procedures described previously [27], [28]. Homozygous progeny plants (T2) were identified, by successive rounds of selection in kanamycin containing (50 µg/l) medium and molecular analysis..

### 2.3 Molecular characterization of transgenic plants

DNA was isolated from putative transgenic plants as described elsewhere [29], and analyzed by PCR, with the same primer pair indicated in the previous section. Reaction mixture was prepared using 10 pmol/µl of each primer, 0.2 mM dNTP, 1.5 mM MgCl_2_ and 2.5 U Taq (Invitrogen). The transgene expression was checked by Reverse Transcriptase PCR (RT-PCR) and Western blot analysis. Total RNA was prepared from transformed leaves by phenol/chloroform extraction, followed by LiCl precipitation, and treated with RNase-free DNase I Amplification Grade (Invitrogen) to remove residual genomic DNA. First-strand cDNA was synthesized using Superscript II (Invitrogen), according to manufacturer's protocol. The amplification of the cDNA region coding for EF-1α gene, an ubiquitously expressed gene [30], was performed as control of cDNA synthesis.

For Western blot analysis, total proteins were isolated from 0.5 g of tobacco leaves, finely powdered in liquid nitrogen and suspended in 300 µl of extraction buffer (6 M urea, 50 mM Tris-HCl pH 7.5, 50 mM NaCl, 5 mM EDTA pH 8). Extracts were centrifuged at 18,800×g, at 4°C, for 20 min to separate the supernatant, containing soluble proteins, from cell debris. Protein concentration was determined by the Bradford method [31], using bovine serum albumin as standard. Total soluble proteins extracted from transgenic and untransformed plants were separated by SDS-PAGE on a Mini-Protein II mini-gel apparatus (Bio-Rad), using 6% (w/v) stacking polyacrylamide gel and 12% (w/v) separation gel. Separated proteins were transferred onto nitrocellulose membrane by electroblotting, with Mini Trans-Blot Cell (Bio-Rad). The blot was probed with the polyclonal antibody anti-c-Myc (A-14) (Santa Cruz Biotechnology), as a primary antibody (dilution 1∶500), and anti-rabbit IgG conjugated with peroxidase (Santa Cruz Biotechnology) as a secondary antibody (dilution 1∶2500). Immunopositive protein bands were visualized through a chemiluminescent detection system (ECL, GE Healthcare), using Hyperfilm ECL (GE Healthcare), and their molecular mass was estimated through comparison with PageRuler Plus Prestained Protein Ladder (Fermentas). The number of transcripts of *Tn*BV*ank1* gene was assessed by Quantitative Real Time PCR (qRT-PCR), targeting a small cDNA region of 85 bp (Forward: 5′-AATGCACCCAACCAAACT-3′, Reverse: 5′-AGCACAGCCATTTCGCCA-3′), and using the actin gene as endogenous control (Forward: 5′-AGGGTTTGCTGGAGATGATG-3′, Reverse: 5′-CGGGTTAAGAGGTGCTTCAG-3′). Reaction mixture was prepared with 300 mM of each primer and 1X Syber Green Master Mix (Qiagen), using the following thermal cycle: 94°C 10 min; 94°C 30 sec, 58°C 30 sec, 72°C 20 sec, repeated 40 times. Serial dilutions of pDE::SP-ank-Myc-KDEL plasmid were used to obtain a standard curve. Fluorescence data were analyzed using Rotor Gene 6000 software Series 1.7.

### 2.4 *S. littoralis* rearing and feeding bioassay

*S. littoralis* larvae were reared on artificial diet [32], at 25±1°C, 70±5% R.H, and under a 16∶8 h light/dark period. The feeding bioassay was performed, under the same environmental conditions, in trays with wells (Bio-Ba-32, Color-Dec, Italy) covered by perforated plastic lids (Bio-Cv-4, Color-Dec Italy). In each well, 3 ml of 1.5% agar agar (w/v), supplemented with 0.005% methyl-p-hydroxybenzoate, were dispensed, to create a moist environment required to keep turgid the experimental tobacco leaves, on which 150 newly hatched larvae were deposited and allowed to develop up to fully grown 2^nd^ instars. Then, soon after their molting to 3^rd^ instar, 64 larvae, for each transgenic line and control plants, were singly transferred into new trays prepared as above, and offered with leaf disks (1 cm^2^) obtained from sub-apical leaves of 4 weeks-old plants, which were daily replaced. At the beginning of the 5^th^ instar, surviving larvae were singly transferred into larger wells (Bio-Ba-8, Color-Dec, Italy) covered by perforated plastic lids (Bio-Cv-1, Color-Dec Italy), and offered with leaf disks 5 cm^2^ in size, which were daily replaced. The survival rate and weight of the larvae were assessed every 24 h, until pupation.

### 2.5 *In vivo* immunodetection of *Tn*BVANK1 and Western blot analysis on insect haemolymph

5^th^ instar *S. littoralis* larvae, reared on leaf disks of tobacco plants as indicated above (section 2.4), were anesthetized by immersion in water, dried on filter paper and cut transversally, to isolate the body segment between the 2^nd^ pair of legs and the 3^rd^ pair of pseudo-legs. The samples were fixed in 4% paraformaldehyde in phosphate buffered saline (PBS), at pH 7.4, for 3 h at room temperature, rinsed 3 times in PBS, dehydrated in an ethanol series and embedded in paraffin. Sections (8 µm thick) were cut with a microtome, de-waxed, using xylene, and rehydrated in an ethanol series.

Sections were rinsed 3 times in PBS, incubated for 30 min with a solution of 2% BSA, 0.1% Tween 20 in PBS (BT-PBS) and then treated with 3% H_2_O_2_ for 10 min, to inhibit endogenous peroxidases. Samples were incubated for 1 h with the primary polyclonal antibody anti-c-Myc (A-14) (Santa Cruz Biotechnology), diluted 1∶50 in BT-PBS. After 3 rinses in PBS, sections were incubated with an anti-rabbit peroxidase-conjugated secondary antibody (Jackson Immunoresearch) (diluted 1∶100 in BT-PBS), for 1 h, and rinsed again in PBS. A DAB (3,3′-diaminobenzidine tetrahydrochloride) substrate was then used to detect the secondary antibody. Negative controls, in which the primary antibody was omitted, did not show any signal. Reference sections were stained with hematoxylin and eosin and examined under a light microscope.

For Western blot analysis of larval haemolymph, 5^th^ instar larvae, reared on leaf disks of tobacco plants as indicated above (section 2.4), were anesthetized by immersion in water and dried on filter paper. Haemolymph was then sampled by cutting off a leg and collecting the exuding haemolymph with a micropipet. Haemolymph samples were mixed with equal volumes of MEAD Buffer (98 mM NaOH, 145 mM NaCl, 17 mM EDTA, 41 mM citric acid, pH 4.5) to avoid melanization. The samples were then centrifuged (1,500×g, 10 min at 4°C) and supernatants stored at −80°C. Prior to use, samples of haemolymph were pooled and concentrated as needed (Concentrator 5301, Eppendorf), dissolved in sample buffer and separated on 12% SDS-PAGE gels. Proteins were transferred onto nitrocellulose membranes with iBlot 2 Dry Blotting System (Life Technologies). Membranes were left for 2 h at room temperature, in 150 mM NaCl, 50 mM Tris·HCl at pH 7.4, 5% w/v nonfat dry milk, 0.1% v/v Tween 20, then were subjected to three washings lasting 15 min each, in BT-PBS, and incubated over-night at 4°C, for 1 h with the primary polyclonal antibody anti-c-Myc (A-14) (Santa Cruz Biotechnology) diluted 1∶1000 in BT-PBS. Membranes were then washed three times (15 min for each washing), and the primary antibody was detected by the enhanced chemiluminescence method (Amersham Biosciences), using peroxidase-conjugated goat anti-rabbit IgG as a secondary antibody (Amersham Biosciences), diluted 1∶16,000. Positope Control Protein (Invitrogen) was used as positive control following manufacturer instructions. Protein concentration of haemolymph samples was determined by the Bradford method [31], using bovine serum albumin as standard.

### 2.6 Cloning expression and purification of *Tn*BVANK1

*Tn*BV*ank1* cDNA was PCR amplified and cloned in NdeI-XhoI site of pET-15b (a kind gift from EMBL, Heidelberg) using site-specific primers: Forward: 5′-CGCGCGCATATGGAAAACTCATTACTCATTGAATTG-3′ and Reverse: 5′-CGCGCGCTCGAGTTACTAATTATCATCACACTTAGCGCCA-3′. The generated plasmid was checked by sequencing and appropriate digestion with restriction enzymes. The recombinant construct was expressed in *E. coli* BL21 (DE3) cells, 16 h at 22°C, in presence of 0.1 mM IPTG (Isopropil-β-D-1-tiogalattopiranoside). After centrifugation (20 min at 4°C at 2,795×g), the pellet was lysed in 20 mM sodium phosphate, 50 mM imidazole, 500 mM NaCl, pH 7.4, 0.2% Triton X-100, in presence of 1 mM phenylmethanesulfonyl fluoride, 5 µg/ml DNaseI, 0.1 mg/ml lysozyme and 1X protease inhibitors (Sigma-Aldrich). Cells were disrupted by sonication and after centrifugation (30 min at 4°C at 21,912×g), protein was purified by FPLC, using an ÄKTA system on a 1 ml His Trap FF column (GE Healthcare), by stepwise elution, according to manufacturer's instruction (GE Healthcare). After elution, *Tn*BVANK1 was dialyzed either in buffer 20 mM sodium phosphate, 300 mM imidazole, 300 mM NaCl, 2 mM DTT, 1 mM EDTA, pH 7.4 (phosphate buffer), or 20 mM piperazine, 20 mM NaCl, 5 mM DTT, 2 mM EDTA, pH 9.8 (pip buffer). Protein purity was assessed on 15% SDS-PAGE gels, using Biorad Precision Plus Protein All Blue Standards (10–250 kDa) as molecular mass marker.

### 2.7 Circular dichroism

Circular dichroism (CD) spectra were acquired as previously described [33]. Briefly, CD spectra were recorded with a Jasco J-715 spectropolarimeter equipped with a Peltier temperature control system [Model PTC-423-S]. Molar ellipticity per mean residue, [θ] in deg cm^2^ × dmol^−1^, was calculated from the equation: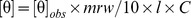, where 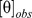 is the ellipticity measured in degrees, mrw is the mean residue molecular mass, C is the protein concentration in mg/ml, and l is the optical path length of the cell in cm. Far-UV measurements (190–260 nm) were carried out at 20°C, at time constant of 4 s, 2 nm band width, scan rate of 20 nm/min, using a 0.1 cm optical path length cell and a protein concentration of 5 µM diluting in water dialyzed samples. CD spectra were signal averaged over at least three scans, and baseline was corrected by subtracting a buffer spectrum.

### 2.8 Dynamic light scattering

Dynamic light scattering (DLS) results were obtained using a Malvern nano zetasizer (Malvern, UK). Samples (0.8–1.5 mg/ml) were placed in a disposable cuvette and held at 25°C during analysis. Each sample was recorded six times with 11 sub-runs using the multimodal mode. The Z average diameter was calculated from the correlation function using the Malvern technology software.

### 2.9 Midgut isolation, preparation of BBMV and transport experiments

The experimental larvae were reared on artificial diet under the environmental condition indicated above (section 2.4). Midguts were isolated from actively feeding 6^th^ instar *S. littoralis* larvae and stored in liquid nitrogen until their use. BBMV (brush border membrane vesicles) were prepared from midguts by Ca^2+^ precipitation and differential centrifugation [34]. The tissue was homogenated in 100 mM mannitol, 10 mM Hepes-Tris, pH 7.2 (10 ml/g tissue). Pellets obtained after the second centrifugation and the final pellets were resuspended in pip buffer (see 2.6) or phosphate buffer (see 2.6). The BBMV protein concentration, estimated with the Coomassie Brilliant Blue G-250 (Thermo Scientific) protein assay, with bovine serum albumin as standard, was adjusted to a final concentration of 5.5 mg/ml. Prior to transport experiments, BBMV were pre-incubated 30 min at room temperature with recombinant *Tn*BVANK1 in the same buffer in which BBMV were resuspended (pip or phosphate buffer), at different experimental concentrations, while control vesicles were pre-incubated with buffer only.

Arginine transport experiments were performed in triplicate at room temperature by rapid filtration under vacuum [34]. The uptakes were measured at 1 min, by mixing 20 µl of the vesicle suspension to 20 µl of the radiolabeled incubation medium, whose final composition was: (i) 20 mM piperazine, 20 mM NaCl, 5 mM DTT, 2 mM EDTA, pH 9.8, 0.05 mM ^3^H-L-arginine 30 µCi/ml, for the BBMV pre-incubated with *Tn*BVANK1 in pip buffer, or with pip buffer only as control; (ii) 20 mM sodium phosphate buffer, 300 mM NaCl, 300 mM imidazole, 5 mM DTT, 2 mM EDTA, pH 7.4, 0.05 mM ^3^H-L-arginine 30 µCi/ml, for the BBMV pre-incubated with *Tn*BVANK1 in phosphate buffer, or with phosphate buffer only as control. Samples were counted for radioactivity in a scintillation spectrometer (Tri-Carb, Packard, model 300 C).

To determine the amount of arginine uptake due to unspecific binding of the labeled substrate and/or to a non-carrier mediated uptake, 0.05 mM arginine uptake into *S. littoralis* BBMV was measured at 11 min, in presence of a 100-fold excess of the cold amino acid. The residual uptake was routinely subtracted from total uptakes.

### 2.10 Statistical analysis

Normality of data was checked with Shapiro-Wilk test and Kolmogorov-Smirnov test, while homoscedasticity was tested with Levene's test and Barlett's test. Survival curves of *S. littoralis* larvae fed on different diets, were compared by using Kaplan-Meier and log-rank analysis. One-Way ANOVA test and Tukey's posthoc test were used to compare larval stage duration and pupal weight. Student's *t* test was used to compare *ank1* transcript levels in transgenic plant tissues and to evaluate the effect of recombinant *Tn*BVANK1 on amino acid transport in BBMVs. All data were analyzed using Prism software, v. 5.0c (GraphPad software; San Diego, California, USA).

## Results

### 3.1 Transgenic plant lines expressing different levels of *Tn*BVANK1

**Figure 1:**
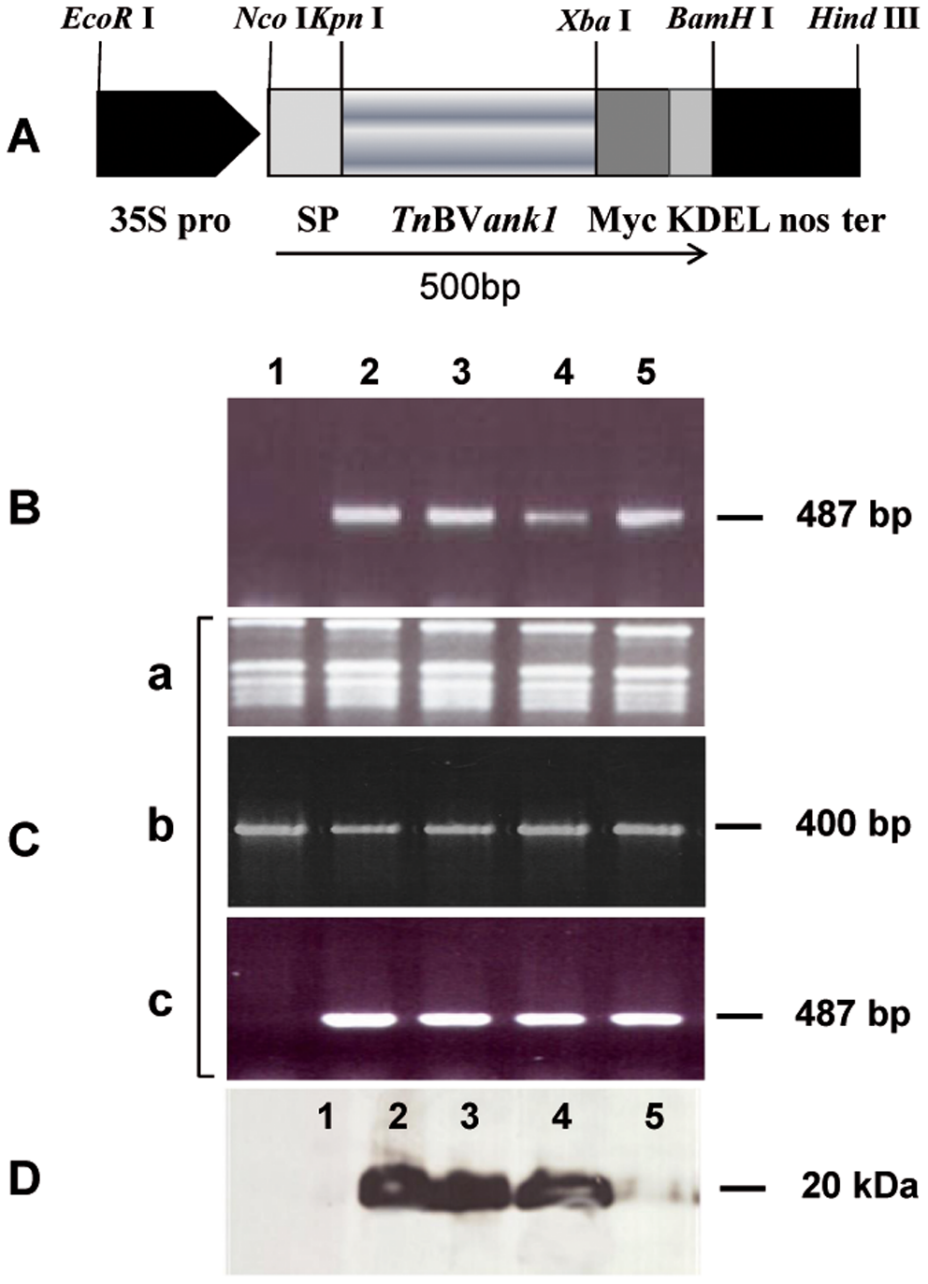
Transgenic plant production and characterization. (A) Schematic representation of the *Tn*BVank1 expression cassette used for the stable genetic transformation. Cis-controlling elements are filled in black and coding sequences are in grey. The restriction enzymes used for cloning are indicated. 35S pro: 35S RNA CaMV gene promoter; SP: sequence coding for the signal peptide of the tobacco *PR1a* gene; *Tn*BV*ank1* sequence of *Tn*BV coding for the viral ankyrin protein used; Myc: c-Myc epitope; KDEL: sequence coding for the endoplasmic reticulum retention signal; nos ter: nopaline synthase terminator sequence. (B) agarose gel electrophoresis of *TnBVank1* amplification products from DNA of putative transgenic plants. (C) RT-PCR analysis of the transgenic lines: (a) agarose gel electrophoresis of total RNA; (b) agarose gel electrophoresis of EF amplicon as quality control of synthesized cDNA; (c) agarose gel electrophoresis of the RT-PCR *Tn*BV*ank1* transcripts confirming the expression of the transgene. (D) Western blot analysis of the proteins from transgenic plant leaves. Lane 1:control plants, lane 2–5: transgenic lines.

**Figure 2:**
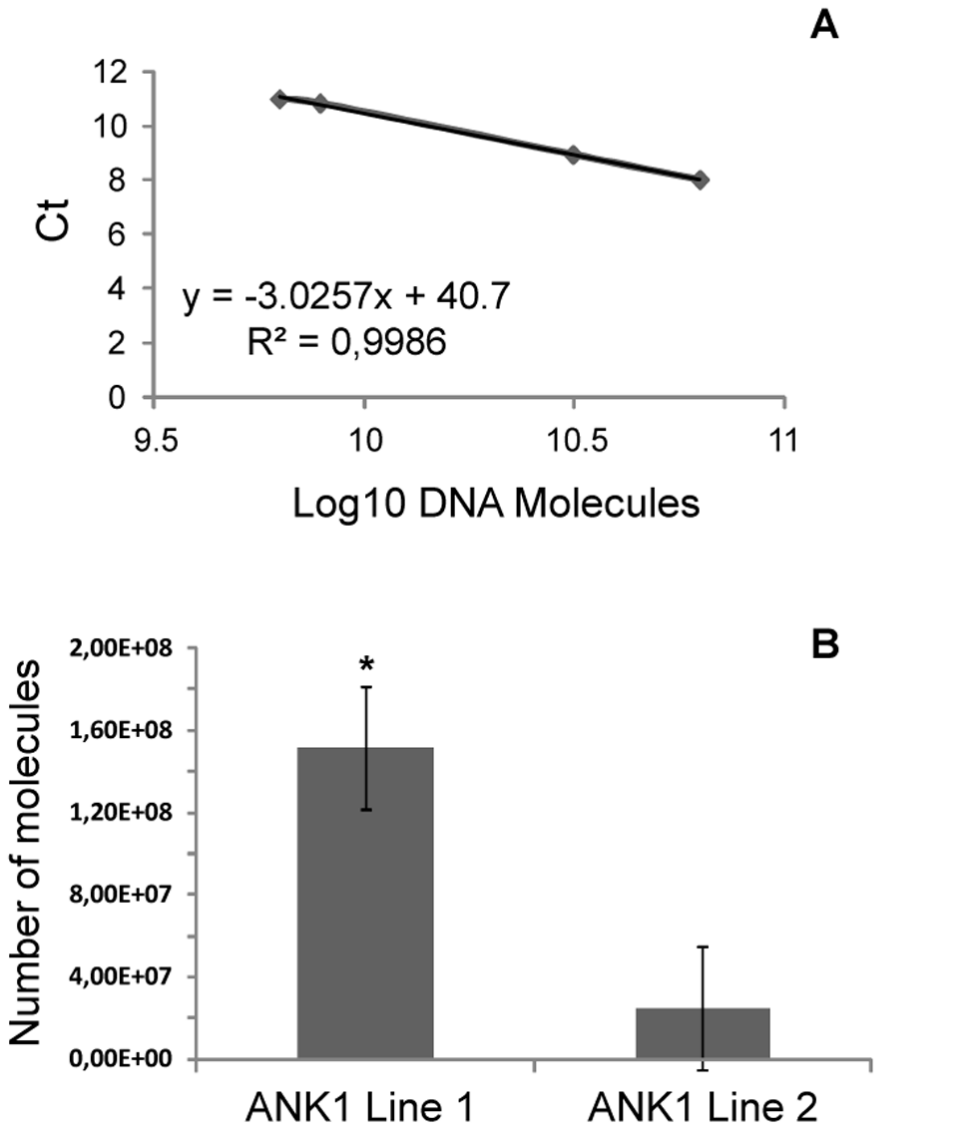
*Tn*BV*ank1* transcription in different transgenic plant lines. The absolute number of transcripts was assessed by qRT-PCR. (A) Standard curve based on serial dilution of pDE::SP-ank-Myc-KDEL plasmid. (B) Number of *Tn*BV*ank1* transcripts in two transgenic genotypes, ANK1 Line 1 and ANK1 Line 2. Mean values denoted with asterisks are significantly different (*P<0.0001).

*Tn*BV*ank1* gene, which codes for a 155 amino acid protein (17.5 kDa), carrying 3 ankyrin domains (amino acids 54–86; 91–121; 125–154), was cloned in the expression cassette represented in Figure 1A. For the immunological detection of the recombinant protein in transgenic plants, *Tn*BV*ank1* cDNA was fused at 3′end to the c-Myc epitope sequence (EQKLISEEDL), that is recognized by a commercially available polyclonal antibody [35]. Moreover, in order to localize the transgenic product in the endoplasmic reticulum, the 5′ end of the chimeric sequence was fused to the Signal Peptide (SP) of the tobacco *PR1a* gene, and the 3′end to the KDEL sequence [36]. The chimeric gene was engineered under the control of CaMW 35S promoter and nos terminator, and the expression cassette was used to transform *N. tabacum cv Samsun*, NN genotype, via *A. tumefaciens*. Putative transformants were screened by PCR, and transgene expression was verified by RT-PCR and western blot analysis (Figure 1B-D). Figure 1B shows PCR amplification of a region of *Tn*BV*ank1* gene, which produced the expected amplicon (size 487 bp). The same pair of primers was used for RT-PCR, normalized on EF-1α gene, which allowed to assess *Tn*BV*ank1* transcription in all transgenic lines considered (Figure 1C). Western blot analysis showed the accumulation of *Tn*BVANK1 recombinant protein in transgenic plants, while no signal was detected in untransformed control plants, as expected (Figure 1D). The molecular mass of the recombinant *Tn*BVANK1 protein was around 20 kDa, indicating that the signal peptide was removed from the mature protein. Two lines producing different amounts of the recombinant protein (Figure 1 D, lanes 2 and 5) were designated as ANK1 Line 1 and ANK1 Line 2, respectively, and used for the feeding bioassay with *S. littoralis* larvae. For these two lines, the absolute number of *Tn*BV*ank1* transcripts was assessed by qRT-PCR (Figure 2). Serial dilutions of pDE∶SP-ank-Myc-KDEL plasmid, containing the whole expression cassette, were used to obtain a standard curve, whose linear equation is y = −3.0257x+40.77 (R^2^ = 0.99) (Figure 2A). This curve was suitable to intersect the Ct value obtained for each cDNA sample, to estimate the number of the molecules [37]. The two transgenic genotypes under investigation showed different amounts of *Tn*BV*ank1* transcripts, which were significantly higher in ANK1 Line 1 (*P*<0.001; *P* = 0.00013, n = 10).

### 3.2 Insecticidal effect of transgenic lines on *S. littoralis* larvae

**Figure 3:**
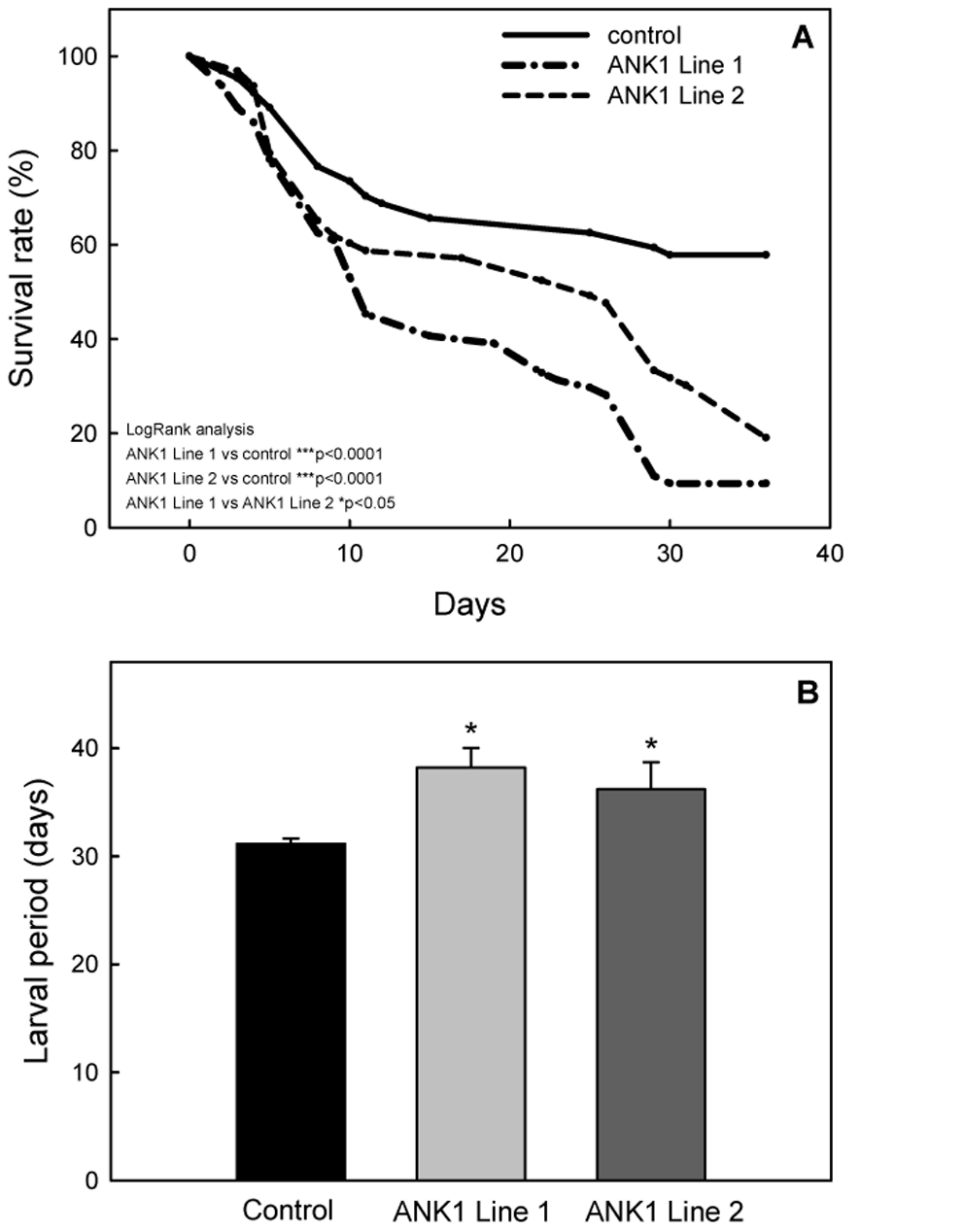
Feeding bioassay with *S. littoralis* larvae. (A) The survival of *S. littoralis* control larvae was significantly higher than in larvae fed on transgenic plant tissues, which displayed a significant difference, positively associated with the transgene transcription level. (B) Duration of larval development for larvae reared on control and transgenic plants. Average values were calculated for larvae surviving at the end of the experiment (control n = 37, ANK1 Line 2 n = 9, ANK1 Line 1 n = 5; *P<0.001) (One way ANOVA; *P* = 0.0001, *F* = 14.790, n = 51).

*S. littoralis* larvae, alimented with leaf disks obtained from leaves of 4 weeks-old plants of ANK1 Line 1 and ANK1 Line 2, showed a significantly lower survival, compared to controls (LogRank test, *P*<0.0001) (Figure 3A). Moreover, experimental larvae fed on ANK1 Line 1, which more intensely transcribed the viral transgene (Figure 2B), in agreement with the higher protein levels observed by Western blot analysis (Figure 1D), showed a significantly more pronounced mortality compared to ANK1 Line 2 (LogRank test, *P*<0.0001) (Figure 3A). After 36 days of feeding, the survival rates were 9.4%, for ANK1 Line 1, 18.8% for ANK1 Line 2 and 57.3% for control.

Larvae and pupae did not show any significant difference in terms of weight gain (Figure S1), however the larval development was significantly delayed for both transgenic lines, compared to controls (control - n = 37, ANK1 Line 2 - n = 9, ANK1 Line 1– n = 5; *P<0.001, One way ANOVA; *P* = 0.0001, *F* = 14.790, n = 51) (Figure 3B); this indicates the occurrence of compensatory feeding on a sub-optimal food source.

### 3.3 Immunodetection of *Tn*BVANK1 in larvae alimented on transgenic plants

**Figure 4:**
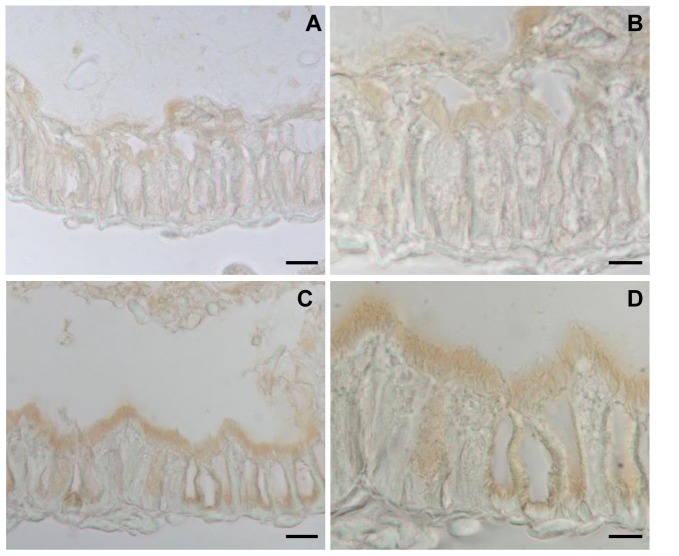
Immunolocalization of *Tn*BVANK1 in the midgut of *S. littoralis* larvae. In samples obtained from larvae fed on control plants, only a faint hybridization signal is visible (A and B), while an evident positive signal is present on the brush border lining the larval midgut epithelium (C and D) of larvae fed on ANK1 Line 1 plants. Bars: A, C 20 µm; B, D 10 µm.

To assess the presence of *Tn*BVANK1 in the tissues of larvae fed with ANK1 Line 1 plants, immunostaining experiments were performed by using an anti-Myc antibody. Although in the midgut of larvae alimented on control plants a faint signal was present (Figure 4A, B), an unexpected strong immunopositivity was detected in the midgut of larvae maintained on transgenic plants (Figure 4C, D). In particular a clear signal was found in the microvillar region of both columnar and goblet cells. No signal was detected on other organs localized in the haemocoel (Figure S2). This evidence was complemented by the absence of hybridization signals on Western blots of haemolymphatic proteins, extracted from experimental larvae alimented with ANK1 Line 1 plants (Figure S3). Collectively, these results indicate that the ingestion of *Tn*BVANK1 is not followed by its transepithelial transport across the midgut, and most of the protein and of its tagged degradation products can be found only in the intestine lumen, where they bind to the apical membranes of midgut cells.

### 3.4 Biochemical characterization of recombinant *Tn*BVANK1

**Figure 5:**
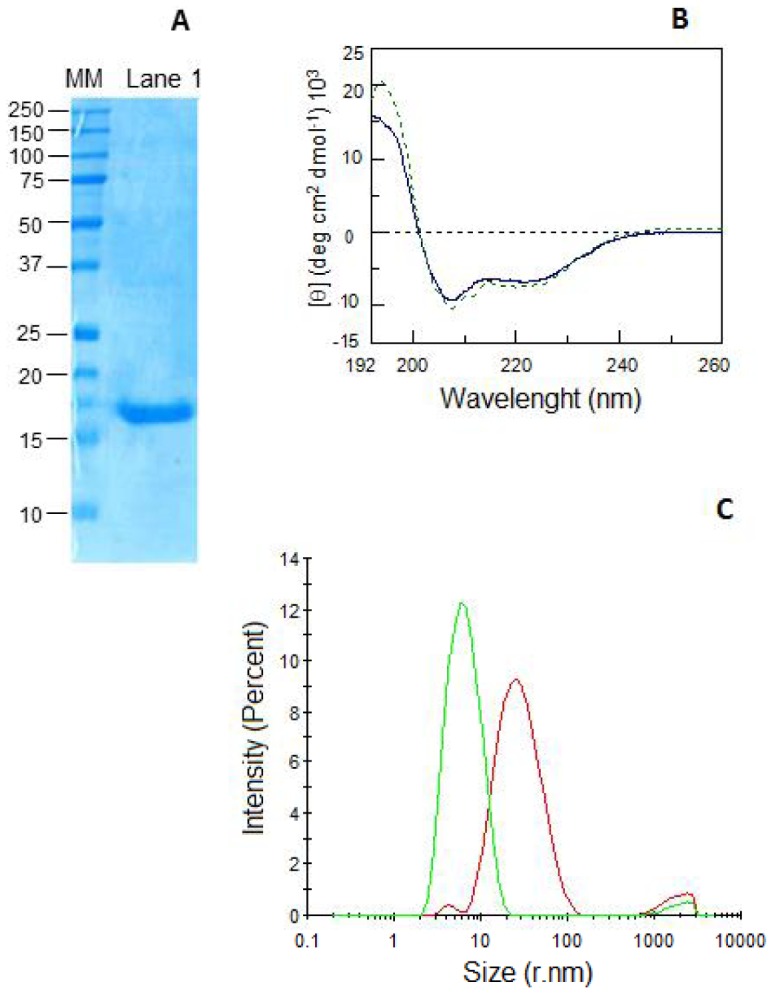
Production and characterization of recombinant *Tn*BVANK1. (A) *Tn*BVANK1 purified by immobilized metal ion affinity chromatography and visualized by Coomassie blue staining of 15% SDS-PAGE gels; MM: molecular mass markers (10–250 kDa), lane 1 *Tn*BVANK1. (B) CD spectra of *Tn*BVANK1 in pip buffer (continuous line) and in phosphate buffer (dashed line). (C) DLS spectra in phosphate buffer (green line) and pip buffer (red line), which indicate the occurrence of different levels of protein aggregation.

To carry out functional experiments aiming to define the possible effects of *Tn*BVANK1 on *S. littoralis* larvae, we produced the recombinant protein in *E. coli*. Bacterial expression of *Tn*BVANK1 and its purification by immobilized metal ion affinity chromatography allowed us to obtain high yields of pure protein (Figure 5A). CD spectra recorded using dialyzed samples showed that *Tn*BVANK1 is in a folded state, displaying a predominant alpha-helix secondary structure (Figure 5B), according to the variable selection method (CDSSTR), using DICHROWEB [38]. The protein was characterized by means of DLS, which allows the measurement of the apparent hydrodynamic diameter [39]. The results showed the occurrence of a unique peak, representative of *Tn*BVANK1 in phosphate buffer (Figure 5C, green curve), which is indicative of a population with a hydrodynamic radius of 5.8±2.2 nm. On the contrary, a notable reduction in intensity of the equivalent peak of *Tn*BVANK1 in pip buffer was observed, whilst the most intense peak shifted to the right, indicating an increase in particle size (Figure 5C, red curve), due to a higher level of protein aggregation.

### 3.5 Effect of recombinant *Tn*BVANK1 on amino acid transport in BBMV

**Figure 6:**
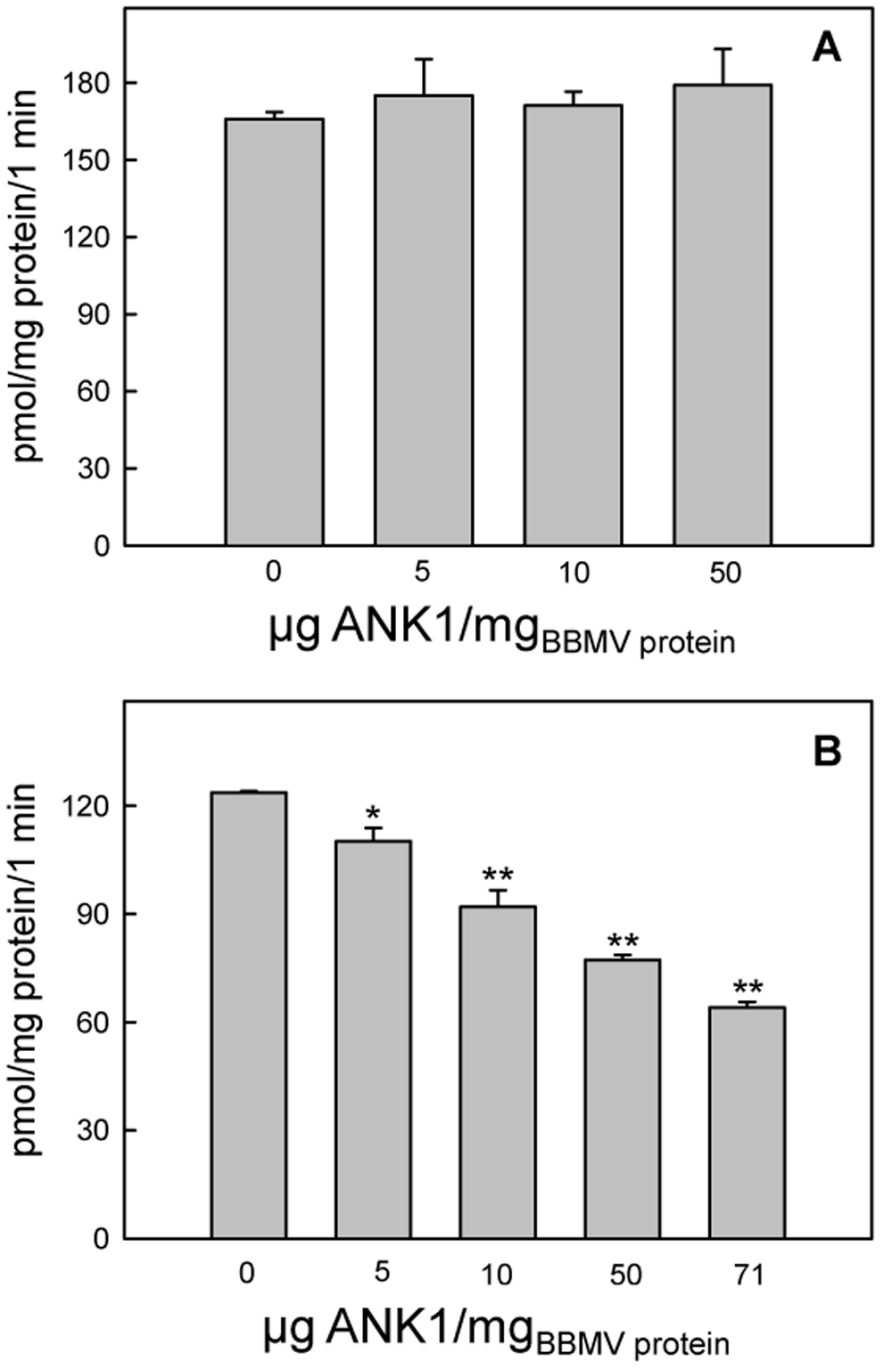
Effect of different doses of *Tn*BVANK1 on arginine uptake in *Spodoptera littoralis* BBMV (Brush Border Membrane Vesicles). (A) Different quantities of BBMV (expressed as mg of proteins) resuspended in pip buffer (see section 2.9 for details) were pre-incubated for 30 min, without (control) or with *Tn*BVANK1, in the same buffer in which BBMV were resuspended. For transport experiments preincubated BBMV were diluted 1∶1 with a cocktail containing radiolabeled arginine, to obtain the final composition reported in section 2.9. (B) BBMV resuspended in phosphate buffer (see section 2.9 for details) were pre-incubated for 30 min, without (control) or with *Tn*BVANK1, in the same buffer in which BBMV were resuspended. For transport experiments pre-incubated BBMV were diluted 1∶1 with a cocktail containing radiolabeled arginine, to obtain the final composition reported in section 2.9. Values are means ± standard error of a typical experiment performed in triplicate. *P<0.02, **P<0.001 (Student's *t* test).

The immunolocalization results and the slower growth of the surviving larvae prompted us to check whether the interaction of *Tn*BVANK1 with brush border membranes (BBM) may impair nutrient transport. Due to the pivotal role of amino acids metabolism in lepidopteran larval midgut [40], these molecules were chosen as model nutrients to study the effect of *Tn*BVANK1 on the transport capacity of columnar cells BBM. The essential amino acid arginine is efficiently transported in lepidopteran midgut, and the functional properties of its transporters, expressed in the apical membrane of columnar cells, have been well characterized in BBMV [41]–[43]. *Tn*BVANK1 in pip buffer, highly aggregated, did not affect arginine transport in our experimental conditions (Figure 6A); on the contrary the lower degree of aggregation observed in phosphate buffer was associated with a dose-dependent inhibition of the amino acid transport (Figure 6B). A significant inhibition of arginine transport was observed with all doses tested and the percentages of inhibition ranged from 11%, in presence of 5 µg *Tn*BVANK1/mg BBMV protein, to 48% with 71 µg *Tn*BVANK1/mg BBMV protein. Collectively, these results indicate that a reduction of the surface interaction between *Tn*BVANK1 and the midgut cells, due to the formation of large molecular aggregates, limits the negative effects that this protein exerts on transport capability of the absorbing epithelium.

## Discussion

The natural antagonism between parasitic wasps and their hosts has generated a wealth of virulence factors used by these insects to overcome multiple defense barriers and to subdue their victims, which are eventually killed [16]. Indeed, these virulence factors originate from an intense co-evolutionary process among an astonishing number of interacting species [14], which has produced one of the largest reservoirs of natural compounds with potential bioinsecticide activity. This molecular biodiversity of parasitic wasps provides an interesting opportunity to develop alternative pest management strategies, based on the use of a new category of natural bioinsecticides, which, so far, have been only limitedly exploited (reviewed in [22]). Here we contribute to this research area, by exploring the impact on lepidopteran larvae of a virulence factor, which is a member of the ANK protein family of the bracovirus associated with *T. nigriceps* [23]. The *ank* gene family is one of the most widely distributed in PDV, both in bracoviruses and ichnoviruses [19]. Moreover, these virulence factors have multiple roles in parasitism, as they disrupt both the immune response and endocrine balance of the host, by interacting with different cellular targets, some of which have been identified through detailed functional analyses [23]–[25], [44], [45]. The pleiotropic effects associated with different viral ANK proteins is likely due to their capacity to establish a number of intense molecular interactions with different targets, often mediated by the ankyrin repeats, typically involved in protein-protein interactions [46]. Therefore, ANK proteins are ideal candidates for the development of new natural bioinsecticides, as, in theory, they are potentially able to impair multiple functions, by hitting different receptors.

We focused our attention on *Tn*BVANK1, a virulence factor involved both in the immune suppression and in the disruption of the ecdysone biosynthesis, when the coding viral gene is expressed in the tissues of parasitized hosts, infected by free virions delivered in the haemocoel at the parasitization, along with the egg and venom [23]–[25]. The idea of testing its possible oral activity was stimulated by previous work, which demonstrated that the expression in plant of Cys-motif proteins, encoded by a polydnavirus or produced by parasitoid-derived cells of embryonic origin (teratocytes), reduced the leaf damage by caterpillar feeding activity [20], [21]. The evidence provided by these studies, however, did not take into account the fate of the parasitoid-derived molecules ingested by the experimental lepidopteran larvae, and did not shed light on how they exerted a negative impact on insect feeding on transgenic plants. Indeed, there was no evidence supporting possible effects on epithelial gut cells of the virulence factor used and/or of its translocation across the gut barrier, to reach the cognate receptors located in the haemocoel. Therefore, any observed biological response induced by these molecules could have been due to their direct effects on the midgut epithelium and/or to their absorption, or to the absorption of their smaller domains resulting from protein digestion.

Here we wanted to assess how the transgenic tobacco plants exert their negative impact on growth and survival of *S. littoralis* larvae and if the expression level of the transgene has a measurable influence. The oral activity of *Tn*BVANK1, which resulted to be dose-dependent, was not associated with any evidence in support of its gut absorption and transepithelial translocation without undergoing enzymatic digestion. Indeed, *Tn*BVANK1 and, possibly, tagged smaller fragments were detected only on the luminal side of the epithelial cells lining the midgut of the experimental larvae alimented with transgenic leaf disks, while no signal was evident in the haemocoel. Therefore, any duplication of parasitism-induced alterations, as a consequence of direct interactions with specific haemocoelic receptors of the entire functional protein, can be reasonably excluded.

The observed uniform and thick layer of immunoreactive material suggested the possibility that the correct functioning of the absorbing epithelial cells of the gut could have been impaired by the presence of this barrier, thus limiting the absorption process and resulting in a kind of starvation effect. This hypothesis has been corroborated by different pieces of experimental evidence we provide in the present study. Indeed, the negative effects on growth and mortality observed were positively associated with different levels of *Tn*BV*ank1* gene expression, in the two transgenic lines tested, suggesting the occurrence of a dose-dependent response. However, more direct evidence in support of this surface interaction underpinning a reduced nutrient absorption is provided by our *in vitro* experiments, where the amino acid transport by BBMV was significantly reduced, in a dose-dependent manner, when incubations were performed in presence of increasing doses of the recombinant *Tn*BVANK1. It is worth noting that this response was not observed when the recombinant protein was dissolved in the pip buffer, which promoted the formation of very large molecular aggregates. This result can be interpreted as a consequence of the fact that reduced surface/volume ratio in larger molecular aggregates reduces the number of exposed interacting domains and generates layers that could be more loosely packed and characterized by lower clotting efficiency. In this case, the observed secondary structure recorded by CD is likely due to the formation of structurally organized aggregates, stabilized by intermolecular interaction [47]. On the contrary, in phosphate buffer *Tn*BVANK1 is not present as a large molecular aggregate but generates smaller structured oligomers, which likely result in a more extensive surface interaction with unknown molecular components present on the apical membrane of absorbing cells, and, thus, negatively affect the efficiency of transport proteins.

Collectively, the experimental data reported indicate that *Tn*BVANK1 is orally active when ingested by larvae of *S. littoralis*, which show slower growth and increased mortality. This is likely induced by the formation of a clotting layer lining the midgut epithelial cells, which reduces the absorption of nutrients, even though we cannot rule out the possible transepithelial effect of untagged bioactive domains, deriving from *Tn*BVANK1 processing. Future studies will have to include the identification of the midgut molecular domains interacting with *Tn*BVANK1, in order to shed light on the mechanisms underpinning the reduced nutrient uptake. In addition, other effects might also contribute to the observed insecticide activity, such as impairment of membrane-bound enzymes in the insect midgut, or plant-mediated effects induced by the expression of the transgene. Indeed, the expression in plants of exogenous proteins with ankyrin domains, which may interact with a number of regulatory proteins, can have unpredictable effects on plant performance and on its defense pathways. These are possible concurring effects, which are worth of future research efforts.

A recent work on lectin mode of action [48] has shown that the ingestion by *S. littoralis* larvae of a plant lectin, from *Hippeastrum* hybrid (Amaryllis) (HHA) bulbs, does not have any toxic effect on isolated midgut cells in culture, but interferes with absorption of nutrients. Circumstantial evidence provided by the same paper indicates that the inhibition of development and the reduced weight gain observed *in vivo* are due to a reduced nutrient absorption, caused by the tight interaction between HHA and the brush border of the midgut epithelial cells. This is fully compatible with growth retardation effects and appears to be quite similar to what observed in *S. littoralis* larvae fed with transgenic plants expressing *Tn*BVANK1. Therefore, in both cases, we may have a starvation-like effect, which, at least in part, may account for the type of negative influence exerted on larval growth and development. This likely hypothesis could be tested by assessing the impact of these two unrelated molecules on gene expression, and by comparing their effect with that induced by starvation. Previous work on *Drosophila* [49] has already indicated that part of the complex transcriptional response elicited by the oral administration of a lectin is similarly induced by starvation, with clear effects on genes involved in carbohydrate metabolism, lipid transport and proteolysis. Therefore, the possibility of making a comparative analysis of the gene expression in *S. littoralis* larvae, as affected by *TnBV*ANK1, HHA and starvation offers new tools and opportunities to investigate the multifaceted entomotoxic effects of lectins, which, to date, are not yet fully understood [50].

The possibility of using molecules of natural origin that do not cause a drastic decline of the target populations has interesting implications from an applied perspective. Indeed, reducing the fitness of phytophagous pests, with insecticide molecules having a moderate impact on their population dynamics, allows to keep in place their antagonists which are part of the higher trophic levels in the natural food-webs. This peculiar aspect, in theory, might help in developing IPM strategies more sustainable from an ecological point of view. However, a careful evaluation of the possible effects that ANK proteins might have on natural antagonists (parasitoids and predators), pollinators and non-target higher organisms is essential to better substantiate their possible future use as novel bioinsecticide molecules.

## Supporting Information

Figure S1 **Feeding bioassay with** ***Spodoptera littoralis*** **larvae.** Larval growth curves and pupal weight were not significantly affected by feeding on transgenic plant tissues compared to controls.
(TIF)

Figure S2 **Immunolocalization of** ***Tn*** **BVANK1 on transverse sections of** ***Spodoptera littoralis*** **larvae.** In transverse sections of larvae fed on ANK1 Line 1 plants, a positive signal is only visible at the brush border of the midgut epithelium. Bar: 150 µm.
(TIF)

Figure S3 **Western blot analysis on** ***Spodoptera littoralis*** **haemolymph.** Haemolymph proteins (15 µg/lane) were separated by SDS-PAGE, on a 12% gel, and analyzed by Western blotting, with polyclonal antibody anti-c-Myc. On the left, nitrocellulose membrane with molecular mass standards (MM in kDa are indicated on the left) and run samples visualized with ponceau dye. On the right, the developed film shows that Myc signal was absent in all samples considered (larvae fed on control tobacco plants - Lane 2, ANK1 Line 1- Lane 3). Tagged Positope reference protein was used as positive control (Lane 1).
(TIF)
