# Supplementary figures and images for: A Virulence Factor Encoded by a Polydnavirus Confers Tolerance to Transgenic Tobacco Plants against Lepidopteran Larvae, by Impairing Nutrient Absorption
Source: PLoS One. 2014 Dec 1;9(12):e113988. doi: 10.1371/journal.pone.0113988 (PMC4250187; doi:10.1371/journal.pone.0113988)

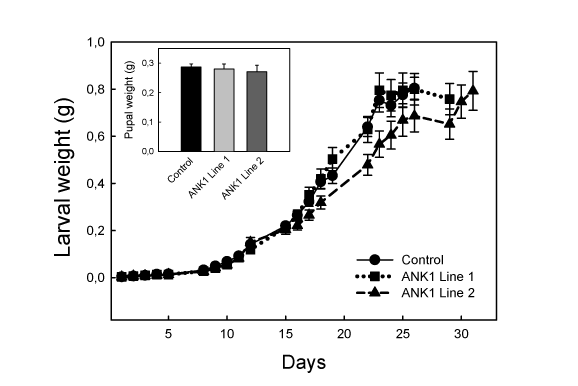

Supplement: Figure S1 — Feeding bioassay with Spodoptera littoralis larvae. Larval growth curves and pupal weight were not significantly affected by feeding on transgenic plant tissues compared to controls. (TIF) [file pone.0113988.s001.tif]

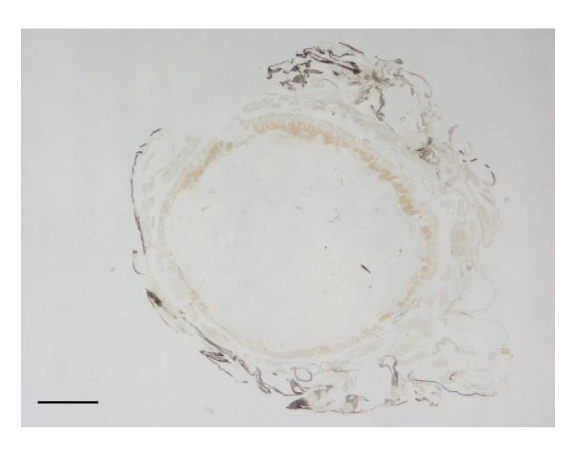

Supplement: Figure S2 — Immunolocalization of Tn BVANK1 on transverse sections of Spodoptera littoralis larvae. In transverse sections of larvae fed on ANK1 Line 1 plants, a positive signal is only visible at the brush border of the midgut epithelium. Bar: 150 µm. (TIF) [file pone.0113988.s002.tif]

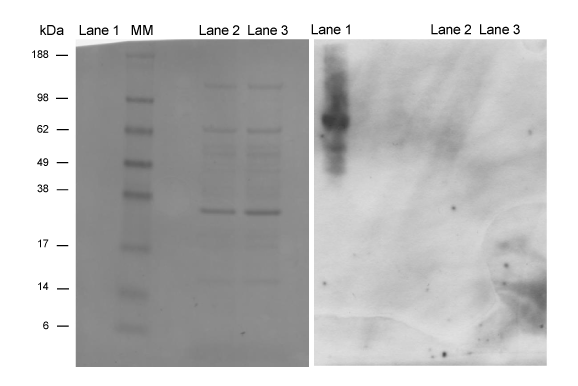

Supplement: Figure S3 — Western blot analysis on Spodoptera littoralis haemolymph. Haemolymph proteins (15 µg/lane) were separated by SDS-PAGE, on a 12% gel, and analyzed by Western blotting, with polyclonal antibody anti-c-Myc. On the left, nitrocellulose membrane with molecular mass standards (MM in kDa are indicated on the left) and run samples visualized with ponceau dye. On the right, the developed film shows that Myc signal was absent in all samples considered (larvae fed on control tobacco plants - Lane 2, ANK1 Line 1- Lane 3). Tagged Positope reference protein was used as positive control (Lane 1). (TIF) [file pone.0113988.s003.tif]
